# Are Keynote and Invited Speakers at State Behavior Analytic Conferences Experts on Their Presentation Topics?

**DOI:** 10.1007/s40617-025-01051-2

**Published:** 2025-04-01

**Authors:** Margaret Ayres Boggs, John T. Rapp, Jennifer L. Cook, Sarah M. Richling, Raeesa Mohamed

**Affiliations:** 1 Department of Psychological Sciences, Auburn University, 226 Thach, Auburn, AL 36849-5214 USA; 2 University of Manitoba, Winnipeg, MB Canada

**Keywords:** Continuing education, Dissemination, Expertise, Publications

## Abstract

Board Certified Behavior Analysts® (BCBA®s) must acquire 32 continuing education units (CEUs) every two years. One way BCBAs obtain CEUs is by attending their state chapter conferences, which feature keynote speakers and invited speakers who disseminate information in their respective areas of presumed scientific expertise. This study evaluated 735 CEU presentations provided by keynote and invited speakers at state conferences in the United States for 2021, 2022, and 2023. For each keynote and invited presentation, researchers used Google Scholar to count the speakers’ (a) peer-reviewed publications on their presentation topic and (b) total peer-reviewed publications. In part, the results across all three years indicate that 31% of speakers had zero topic-specific publications. Notably, the percentage of speakers with zero topic-specific publications concerningly increased across the three years. Results also indicate nearly 40% of speakers with zero topic-specific publications did not have any peer-reviewed publications. We discuss the potential implications of the findings and suggest actions for offsetting the current trend.

Health and service delivery fields, including applied behavior analysis (ABA), typically require their practitioners to participate in continuing education activities to maintain certification and licensure within those fields. The Behavior Analysis Certification Board® (BACB®) handbook describes continuing education units (CEUs) as a way to “stay up to date on the developments of the profession” (BACB, 2025b, p. 40). The *Ethics Code for Behavior Analysts* outlines numerous standards for practitioners, including practicing within one’s scope of competence in a culturally responsive and humble manner, all while making data-based decisions (BACB, 2020). According to the BACB code, each practitioner is responsible for upholding the overarching ethical standards by participating in professional development opportunities and instilling these values in supervisees (BACB, 2020).

CEU activities are grouped into one of three categories described by the BACB: (a) Learning, (b) Teaching, and (c) Scholarship (BACB, 2025b). Among the three methods, most certificants engage with the learning category, which includes synchronous or asynchronous events offered by authorized continuing education (ACE) providers (BACB, 2025b; Kranak, Andzik, et al., 2023). In addition, there are various modalities by which behavior analysts can contact continuing education content. Regardless of the delivery modality, CEU presentations should be empirically based learning opportunities for attendees. Indeed, one of the four Core Principles of the BACB *Ethics Code for Behavior Analysts* describes that behavior analysts must ensure their own competence (BACB, 2020, p. 4); this includes maintaining their knowledge of the science (and the potential harm from pseudoscience) in their practice area(s). Moreover, “Code 2.01 Providing Effective Treatment” requires behavior analysts to rely on scientific evidence, and “Code 1.06 Maintaining Competence” requires their participation in professional development activities, including conference attendance, as one such method.

Board Certified Behavior Analysts® (BCBA®s^1^) and Board Certified assistant Behavior Analysts® (BCaBA®s) must acquire 32 and 20 CEUs on a 2-year cycle, respectively, to retain their certification (BACB, 2025a, 2025b). The BACB allows practitioners to determine the material they want to consume, but it requires certificants to complete four mandatory ethics credits, as well as three supervision credits if they provide supervision (BACB, 2025b). Typically, state licensure requirements for behavior analysts follow the BACB standards (Kranak, Andzik, et al., 2023). During the past decade, the BACB increased the required number and specificity of the CEUs and simultaneously decreased the recertification interval. Beginning in 2026, the BACB will require new certification and maintenance standards for front-line staff who are credentialed as Registered Behavior Technicians®, which includes 12 CEUs per 2-year recertification cycle as one of the new standards (BACB, 2023). To our knowledge, few studies have evaluated where certificants obtain their CEUs.^2^

Kranak, Andzik, et al. (2023) surveyed 231 behavior analysts (primarily consisting of BCBAs) about the conferences and other CEU events they attended. In part, respondents expressed higher trust in content offered by ACE providers during in-person CEU events and webinars than with content provided via podcasts and related media outlets. They also found over 80% of their respondents had attended at least one conference in the past 24 months and that employers did *not* typically reimburse respondents for their CEU expenses. As such, it may be reasonable to assume that practitioners will minimize out-of-pocket expenses when obtaining CEUs by attending state ABA conferences.

State-level behavior analytic conferences host as few as one and as many as 50 invited speakers who present content for CEUs. To qualify as a CEU provider, the ACE Provider Handbook (BACB, 2022) requires state associations to have an authorized ACE organization provider who serves as the ACE Coordinator and either directly or indirectly (i.e., via the presenter) attests to the behavior-analytic nature and quality of each presentation. Most state behavior-analytic conferences offer CEU experiences that include workshops, panel discussions, symposia, and invited presentations. Keynote presentations are a subset of invited presentations wherein the speaker has the added expectation of, loosely speaking, “setting the tone” of the conference. Organizations also rely on keynote speakers to attract and engage as many attendees as possible (Kranak & Onofrio, 2025). Thus, individuals serving in keynote speaker roles should have expertise within the field and their subject area commensurate with their keynote speaker status (Kranak, Andzik, et al., 2023). Typically, a keynote speaker is (or should be) a regionally, nationally, or internationally recognized expert on their presentation topic (Kranak & Onofrio, 2025; Kranak, Hall, et al., 2023). In other words, those with an extensive background on the topic should deliver continuing education instruction, especially keynote presentations. Nevertheless, few studies have evaluated the qualifications of keynote and other invited speakers at state-level behavior analytic conferences.

In one of the few studies on this topic, Kranak, Hall, et al. (2023) examined the representation of women as keynote and invited speakers at state association conferences in behavior analysis from 2015 through 2020. The researchers reviewed programs from 180 state-level conferences, and they identified 172 keynote and 431 invited presentations. Their results generally indicated that fewer women than men served in both roles during the 6-year study period. However, regardless of gender identity, their results indicated that 88% of keynote and 68% of invited speakers had served in the same capacity at multiple state-level conferences in the prior 5-year period. This means that many of the same individuals served as keynote and invited speakers at multiple conferences during 2015–2020. The researchers provided a list of 20 individuals who gave the most combined keynote and invited presentations during the study period. On the basis of our informal review of that list, most, if not all, of the top 20 presenters had published extensively on one or more topics in behavior analysis, practiced ABA for multiple decades, or both. That is, most individuals on that list would likely be considered experts on one or more topics in ABA. However, their speaker list does not indicate the topic of each keynote and invited presentation, so it is unclear whether each presenter was an expert on their presentation topic. In other words, one or more of those individuals may have given presentations on topics for which they had no scientific expertise. Although Kranak, Hall, et al. reported on the most frequent presenters in the timeframe included in their analysis, they did not analyze the extent to which those frequently invited presenters were experts. In the current study, we argue that topic expertise largely refers to peer-reviewed publications, especially contemporary publications (Carpenter et al., 2014; Dixon et al., 2015a, 2015b; Kranak & Onofrio, 2025). To that end, determining a given speaker’s expertise is likely a matter of the match of the presentation topic with the speaker’s research history (e.g., the number of peer-reviewed publications on the topic).

To our knowledge, there is no universal agreement about what constitutes expertise within a given discipline. Thus, evaluators must technologically define expertise and identify corresponding dependent measures. It is reasonable to assume that a speaker offering CEUs on their presentation content should know more about their presentation topic than others in their audience who do *not* present on the given topic. How does one determine if a speaker does, in fact, know more about a given topic than those who sit in their audience? The field of ABA does not have a consensus position on variables that address this question. Given the absence of a formal standard, we argue that the number of peer-reviewed publications on a specific presentation topic and the total number of publications are both face valid measures of a CEU presenter’s level of scientific expertise. Importantly, both metrics are readily available for evaluation by members of the scientific community. Other measures, such as years of clinical experience, may also be pertinent to expertise but are more difficult for the scientific community to quantify. Specifically, because practitioners cannot ethically or legally share the clinical records of their clients, their clinical experience can only be quantified in terms of years of practice. As such, clinical experience cannot be subjected to the same level of scrutiny as peer-reviewed publications.

The peer-review process for publications was designed to uphold the validity and accuracy of the scientific findings (Kelly et al., 2014). Researchers in other disciplines have used publication rates to evaluate presentation content at conferences (e.g., DeMola et al., 2009; Grover & Dalton, 2020). For example, DeMola et al. (2009) found only 321 (57.5%) of the 558 abstracts presented at orthopedic conferences resulted in peer-reviewed journal articles. DeMola et al. expressed concern with their findings because major orthopedic textbooks regularly cited the content of conference presentations, almost half of which were not published, as scientifically accepted material for directing the actions of practitioners. More recently, Grover and Dalton (2020) evaluated 1081 free presentations provided at psychiatry conferences and found only 27% of abstracts resulted in publications within 5 years of the conference. In one of the few studies on content at a behavior-analytic conference, Richling et al. (2014) tracked 61 CEU presentations offered at ABAI in 2005 over a 5-year period and found only 13% of those presentations^3^ had content that gave rise to a peer-reviewed article. Notably, Richling et al. did not distinguish between invited (including keynote) and non-invited presenters.

Although the field of ABA has not yet adopted a system to evaluate CEU presentations at international, national, regional, or state levels, prioritizing the examination of the expertise of keynote and other invited speakers at state-level ABA conferences seems warranted for two broad and related reasons. First, most state organizations hold at least one conference each year, with multiple individuals serving as keynote and invited speakers. Thus, conferences provided by state-level organizations likely contain a high percentage of presenters who provide CEU content. Second, Kranak, Andzik, et al. (2023) found many BCBAs in their sample had attended, and also preferred, in-person conferences for obtaining their CEUs. Interestingly, Kranak, Andzik, et al. also found that many BCBAs did not receive reimbursement from their employers for the CEU events. Although the researchers did not specify whether the virtual and in-person conferences were at the national, regional, or state level, it seems likely practitioners are obtaining at least some of their required 32 CEUs every two years from local state conferences, which often offer six to eight CEUs per day and likely cost less money and time to attend than national conferences. Given that state conferences are likely to be consistent sources of CEU information for local certificants and that state associations take on local leadership roles in the field, evaluating the expertise of keynote and invited speakers who provide CEUs at these conferences is warranted.

The current study endeavored to extend the studies by Kranak and colleagues (2023, 2025) by evaluating the expertise of keynote and invited speakers at state ABA conferences. For each keynote and invited presenter,^4^ we used the number of peer-reviewed articles the speaker published on their presentation topic as our dependent variable for determining their level of expertise. We defined minimal expertise as the speaker having published one or more peer-reviewed articles on their presentation topic. To be included as evidence of the speaker’s expertise, we only required that the speaker was listed as a coauthor of the published articles (i.e., we did *not* require them to be the first author). We deliberately set a relatively low bar for determining a speaker’s minimal expertise via peer-reviewed publications for two reasons. First, given the exponential increase in practicing BCBAs from 2018 to 2023 (BACB, 2024) and the corresponding growth in state conferences, it is reasonable to assume that there are not enough mid- and senior-level researchers to present at each state conference. Thus, junior researchers are a reasonable option to fill this void. Second, if a presenter has one or more recent publications on a given topic, they are likely familiar with the most recent literature on the topic. In this way, they are *probably* more knowledgeable on the topic than people to whom they present. Moreover, state conferences potentially serve as a springboard for junior university faculty to gain relevant experience presenting at professional conferences (Kranak & Onofrio, 2025). Importantly, a presenter in the current study could be considered an expert in their topic without being a BCBA or BCBA-D. For example, a keynote presenter who published 10 studies on token systems with nonhumans could be considered an expert when presenting on token economies for CEUs at a state ABA conference.

We also measured each presenter’s total publication count as a general measure of research productivity as opposed to a specific measure of expertise on a topic. This variable could be an informative supplement to a measure of peer-reviewed publications on the presentation topic. For example, a presenter with no publications on their presentation topic but 60 overall publications may not be a topic expert, but they could be an appropriate presenter of the topic, particularly if they published extensively on a related topic.

When our results indicated presenters had not published articles on either their presentation topic or other topics, researchers collected data on speakers’ alternative experiences, such as their participation in social media events (e.g., podcasts) and their years of providing clinical services. The purpose of collecting data on alternative sources for speakers without sufficient scientific expertise was to evaluate possible factors that led to the selection of these speakers. Kranak and Onofrio (2025) found most state-level organizations in their sample invited their conference speakers on the basis of the speaker’s popularity; however, the researchers did not identify factors that contributed to the speakers’ popularity.

## Method

### Rationale and Participants

**Fig. 1:**
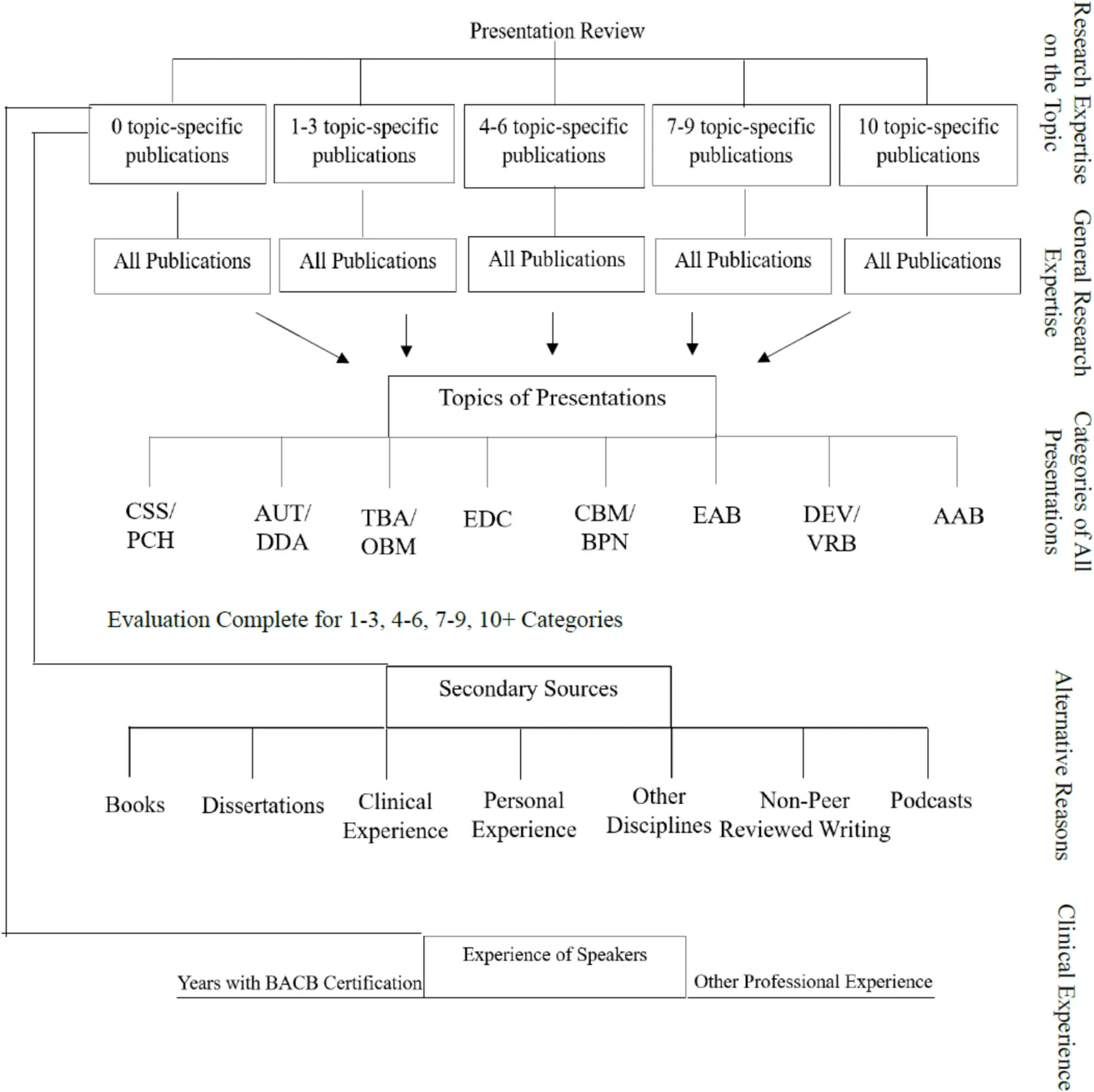
Flowchart of review process for keynote speakers and invited speakers

Prior to beginning the study, we submitted a determination application, which outlined our proposed study, to an institutional review board (IRB). After reviewing the application, the IRB concluded that the study did not require IRB human subjects review.^5^ The data collectors were four undergraduate psychology students and two graduate students in a Master’s ABA program. The first author trained each data collector via Zoom using behavioral skills training until each data collector could independently display all of the search steps as outlined in Fig. 1. Thereafter, the first author met weekly with data collectors and provided booster training sessions as needed. The current study evaluated publications by keynote speakers and invited speakers *near or prior* to the speaker’s presentation date. For example, if an individual presented in 2021 and data collectors identified a topic match in a peer-reviewed article published in 2022, data collectors counted the article as a match with the presentation topic for that presenter. This approach allowed for studies that may have been under peer review when speakers presented.

### Procedures

For the initial phase of data collection, the six data collectors acquired publicly available information about keynote and other invited presentations at state ABA conferences. Thereafter, they gathered information about the keynote and other invited speakers’ publication history and, when necessary (due to inadequate publication records), information about their alternative experiences, such as professional and practice history. Data collection began in April 2023 for all ABAI-affiliated state chapter conferences in 2021, 2022, and 2023, and the study concluded in April 2024. The official ABAI website listed 54^6^ affiliated regional and state chapters. The state chapters were selected for this study. The profile contained a link to the individual websites of each chapter. Because we opted to evaluate conferences at the individual state level, we did not evaluate several regional conferences (i.e., Berkshire Association for Behavior Analysis and Therapy, Four Corners ABA, Southeastern ABA, Mid-American ABA, and Hoosier ABA). We evaluated keynote and invited speakers for conferences that occurred during the years 2021, 2022, and 2023, with the exception of when the organization did not list both the titles of presentations *and* the speaker’s names online. In addition, we attempted to evaluate speakers at Behavior Association of Michigan, Delaware ABA, District of Colombia ABA, Lone Star ABA, Oklahoma ABA, Philadelphia Metropolitan ABA, and West Virginia Behavior Analysis Association, but these chapters did not host conferences during the study period. After applying the aforementioned criteria, the analyses included keynote speakers and invited speakers from 42 organizations.

#### Evaluating Keynote and Invited Presenters

Similar to the methods used by Richling et al. (2014), data collectors extracted information about the keynote and invited presenters from the conference- and organization-related newsletters, web pages, social media pages and posts, and virtual programs. The information needed to complete our evaluation included the (a) names of the keynote and invited speakers, (b) availability of CEUs for the keynote and invited presentations, (c) keynote and invited speaker certification level (i.e., BCBA or BCBA-D), and (d) respective presentation titles. Each ABAI-affiliated chapter website or social media account typically contained information on the conference speakers, keywords to search for more information about their annual conference, or both. When information about conference speakers was *not* publicly available, data collectors emailed board members who were listed on the respective conference websites for the conference programs. Data collectors also used the BACB Certificant Registry, which publicly lists current and expired certifications, to determine (or otherwise verify) (a) whether each speaker was a BCBA or BCBA-D and (b) the year each speaker was first certified.

If speakers gave multiple presentations at a given conference, data collectors counted each CEU opportunity separately. It was possible for a given keynote or invited presenter to be an expert on one presentation topic and have no expertise on another topic at the same conference. In the event that one presentation featured two keynote or invited presenters, data collectors included only the speaker with the most expertise (described below). We *excluded* invited speaker panels, workshops, and invited presentations that did not provide CEUs. In five cases, the conference program contained language that implied (e.g., by referring to “featured” speakers) but did not clearly specify whether their speakers were, in fact, invited presenters. We opted to include these speakers as invited presenters in our evaluation.

#### Primary Measures

Figure 1 outlined the general process data collectors followed for evaluating keynote and invited presentations. The primary dependent measures were each speaker’s number of publications on their presentation topic and each speaker’s overall publication count. Dixon et al. (2015a) evaluated faculty members’ research productivity by counting their publications in behavior-analytic journals using Google Scholar. For the current study, data collectors used Google Scholar to collect data for publications in any peer-reviewed outlet. When necessary, data collectors used ResearchGate to obtain additional information on the speaker (e.g., their affiliation) to confirm that they had evaluated the correct individual from the corresponding conferences.

Each keynote and invited speaker’s presentation title and abstract typically provided enough information to determine keywords regarding the main topic of their CEU presentation. If data collectors could not find the description, they searched Google for the title of the presentation. In most cases, they found an abstract if the speaker (a) provided online CEU content, (b) repeated the presentation at another conference, or both. As a hypothetical example, if they collected data on a presentation titled “Assessment and Treatment of Adolescent Problem Behavior Maintained by Escape,” some keywords could have been “escape” and “problem behavior.” If the abstract mentioned “functional analyses” and “antecedent manipulations,” data collectors would have included these as keywords to refine the search in future steps. In order to provide a liberal evaluation, the publications could have differed from the conference presentation in areas such as title, behavior, design, and procedure. For example, if the speaker presented on feeding disorders, all peer-reviewed publications detailing feeding in the abstract or title counted as “a publication on the topic.”

To determine the number of publications on the presentation topic, data collectors read each title and abstract of the peer-reviewed articles listed on Google Scholar and then manually searched for matches with the presentation topic. If one or more keywords matched a publication and the presenter was the author or a co-author of the article, the data collectors counted that publication toward the speaker’s expertise for that presentation. Data collectors discontinued this search process after they found more than 10 publications on the topic by the speaker. Data collectors then used Google Scholar to determine the total number of publications by the speaker. Conversely, when Google Scholar yielded no articles authored or co-authored by the presenter, data collectors coded the presenter as having (a) no topic-specific published articles and (b) no overall publications.

#### Alternative Experiences

If the initial search process did not yield any topic-specific published articles for a speaker, data collectors searched for alternative experiences on LinkedIn™ and official university websites. We collected data on this broad variable to understand why organizations selected speakers without scientific expertise, as evidenced through publishing, as keynote or invited speakers. If the presenter worked at a university, the university website typically contained their curriculum vitae. Data collectors scored alternative experiences that pertained to the presentation topic, such as (a) authoring books/chapters, newsletters, podcasts, and blogs; (b) clinical and personal experiences (e.g., founder of a company, personal diagnoses, parent of child with diagnoses, volunteer work listed on LinkedIn); and (c) practicing in a related discipline (e.g., speech pathologist, clinical psychologist, lawyer). Specifically, data collectors scored the personal experience category when a presenter self-reported being autistic (in the conference program), *and* the diagnosis was pertinent to the topic of the keynote or invited presentation. For the other experiences, data collectors used the same liberal criteria described above for matching presentations to publications.

For all keynote and invited speakers, data collectors assigned the topic of their presentation to one of the 13 ABAI conference presentation categories (see also Kangas & Vaidya, 2007). As shown in Fig. 1 (bottom), researchers combined ABAI’s broad topics to make eight categories for the current study: Autism/Developmental Disabilities (AUT/DDA), Applied Animal Behavior (AAB), Experimental Analysis of Behavior (EAB), Community, Social, and Sustainability Issues/Philosophical, Conceptual, and Historical Issues (CSS/PCH), Teaching Behavior Analysis/ Organizational Behavior Management (TBA/OBM), Clinical, Family, and Behavioral Medicine/Behavior Pharmacology and Neuroscience (CBM/BPN), Behavioral Development/Verbal Behavior (DEV/VRB), and Education (EDC). Researchers combined ABAI topic categories on the basis of overlapping content of categories, a low count of presentations in two similar categories, or both.

### Intercoder Agreement

We assessed intercoder agreement using procedures similar to those described by Richling et al. (2014). Two data collectors independently coded 34% of the variables on the keynote and invited speakers. Researchers calculated intercoder agreement scores using the average agreement^7^ across presenters for topic-specific publications and total publication count, separately. For each presenter, we divided the smaller topic-specific publication count by the larger topic-specific publication count. We then summed up the values across presenters, divided that value by 250 (the number of observations with a secondary observer), and multiplied the quotient by 100%. We repeated this process for the total publication count with each presenter. Researchers used an exact agreement method for CEU availability, speaker affiliation, presentation title, ABAI topic categorization, and the number of alternative sources. Between the two data collectors, each variable had to contain point-to-point correspondence. Researchers calculated exact agreement scores for each dependent measure by dividing the number of agreements by the number of disagreements plus agreements and multiplying the quotient by 100%. The average intercoder agreement scores for topic-specific publications and total publication count were 83% (range, 43–100%) and 93% (20–100%), respectively. The intercoder agreement scores for affiliation, title, topic categorization, and number of alternative sources were 100%, 100%, 90% (75–100%), and 87% (35–100%), respectively.

### Determining Expertise

After data collectors completed the process described above, we assigned the invited speakers a “level of expertise” rating based on the number of publications that were specific to their presentation topic. We categorized speakers with 0 topic-specific publications as having no scientific expertise on that topic. In addition, we categorized speakers with 1–3, 4–6, and 7–9 topic-specific publications as having low, moderate, and high-moderate expertise, respectively. Notably, if a speaker had only one topic-specific publication, we speculated (but did not verify) that it was likely to be a study they conducted as their thesis or dissertation. To this end, it is likely that the speaker demonstrated their knowledge of their topic to their respective committee members. Moreover, Kranak and Onofrio (2025) noted that multiple organizations used the same keynote and invited speakers within and across conferences and argued that this practice could impede the professional development of junior faculty. For these reasons, we did not view having only one topic-relevant presentation as an inherent problem for a keynote or invited speaker. Finally, we categorized speakers with 10 or more topic-specific publications as having strong expertise on their presentation topic.

## Results

**Fig. 2:**
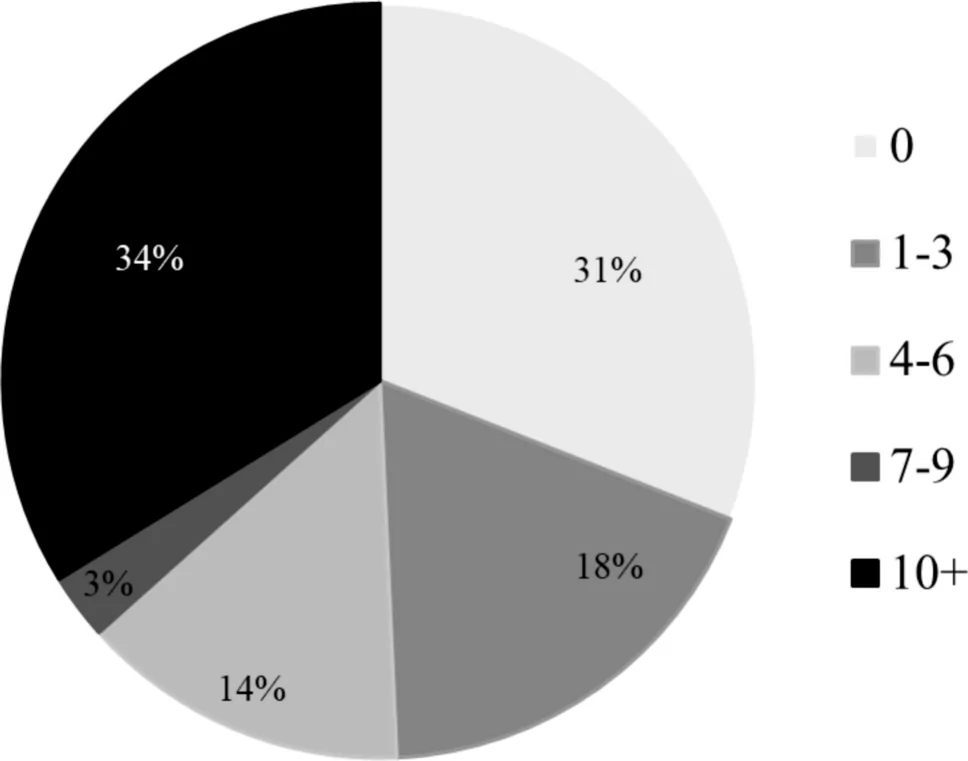
Percentage of keynote speakers with 0 to 10 + topic-specific presentations. *Note*. *n* = 71 keynote presenters. Category 0 = 22 presenters, category 1–3 = 13 presenters, category 4–6 = 10 presenters, category 7–9 = 2 presenters, and category 10 +  = 24 presenters. Percentages were rounded to the nearest whole number

**Fig. 3:**
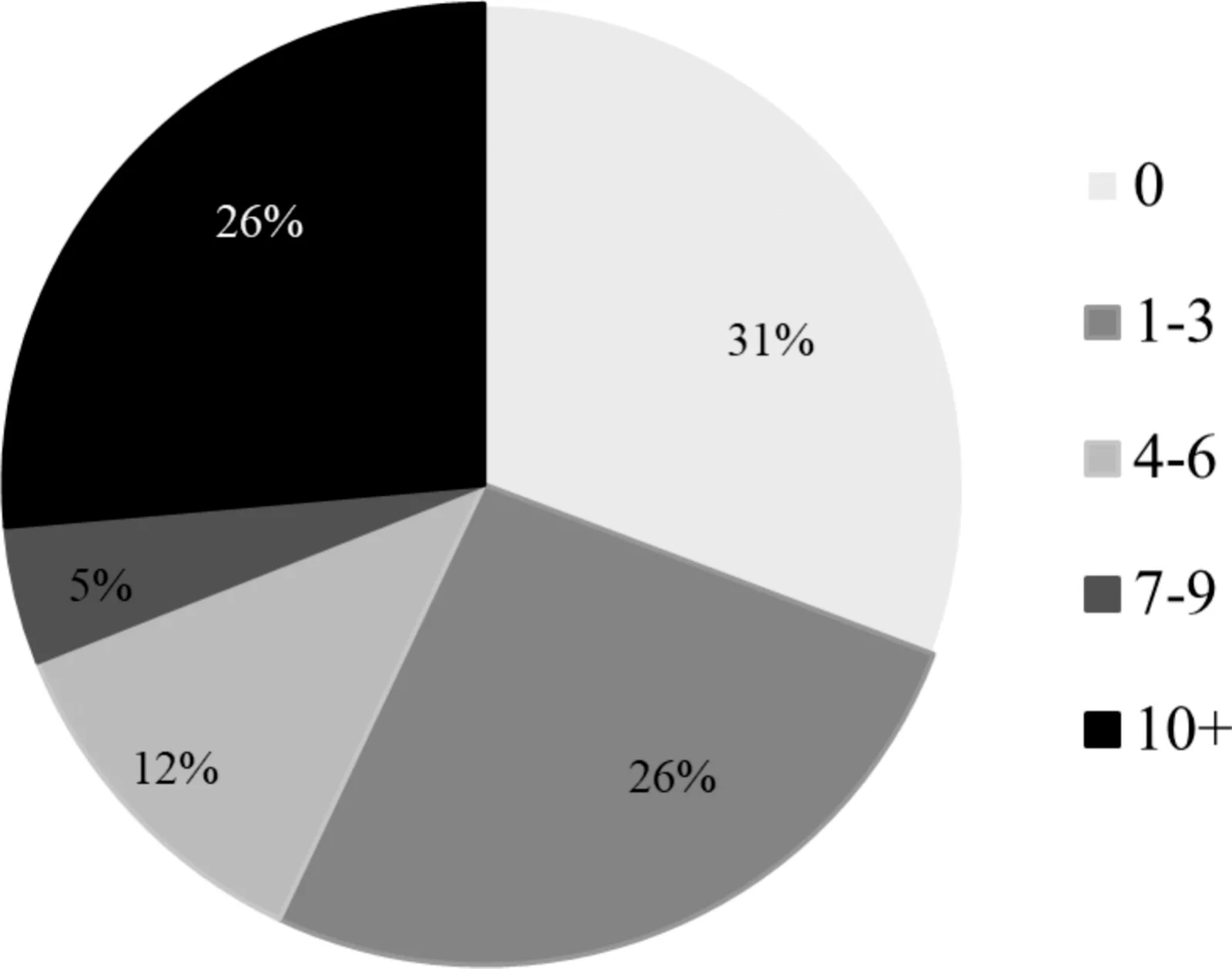
Percentage of all keynote and invited presenters with 0 to 10 + topic-specific presentations. *n* = 735 presentations. Category 0 = 226 presenters, category 1–3 = 193 presenters, category 4–6 = 88 presenters, category 7–9 = 34 presenters, and category 10 +  = 194 presenters. Percentages were rounded to the nearest whole number

Data collectors evaluated 735 invited presentations across 111 conferences. Of the 111 conferences, 34, 38, and 39 were held in 2021, 2022, and 2023, respectively. Figure 2 shows the percentage of *keynote* speakers with 0, 1–3, 4–6, 7–9, and 10 + topic-specific publications. Results show that even though keynote speakers have the highest status at each conference, nearly a third did not have *any* publications on their presentation topic. Moreover, including those with zero topic-specific publications, nearly 50% of all keynote speakers had three or fewer publications on their presentation topic. Figure 3 shows the combined percentage of both keynote speakers and invited speakers with 0, 1–3, 4–6, 7–9, and 10 + topic-specific publications. Results show that 31% of all keynote speakers *and* invited speakers did not have any publications on their presentation topic, and 57% had three or fewer publications on their topic.

**Fig. 4:**
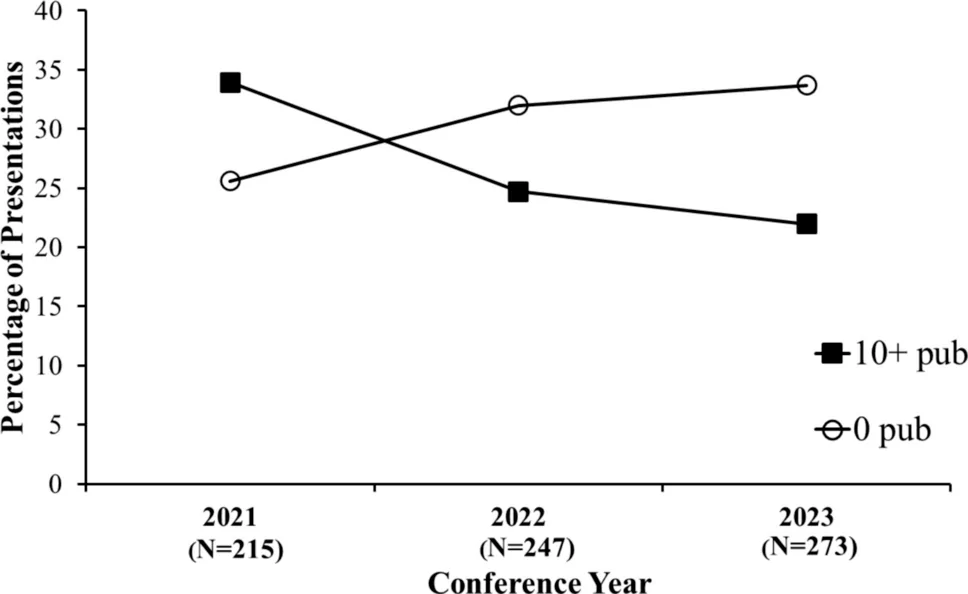
Percentage of speakers with 0 and 10 or more topic specific publications across years. The *Ns* for each year include all five topic-specific publication categories. Pub = topic-specific publication

**Fig. 5:**
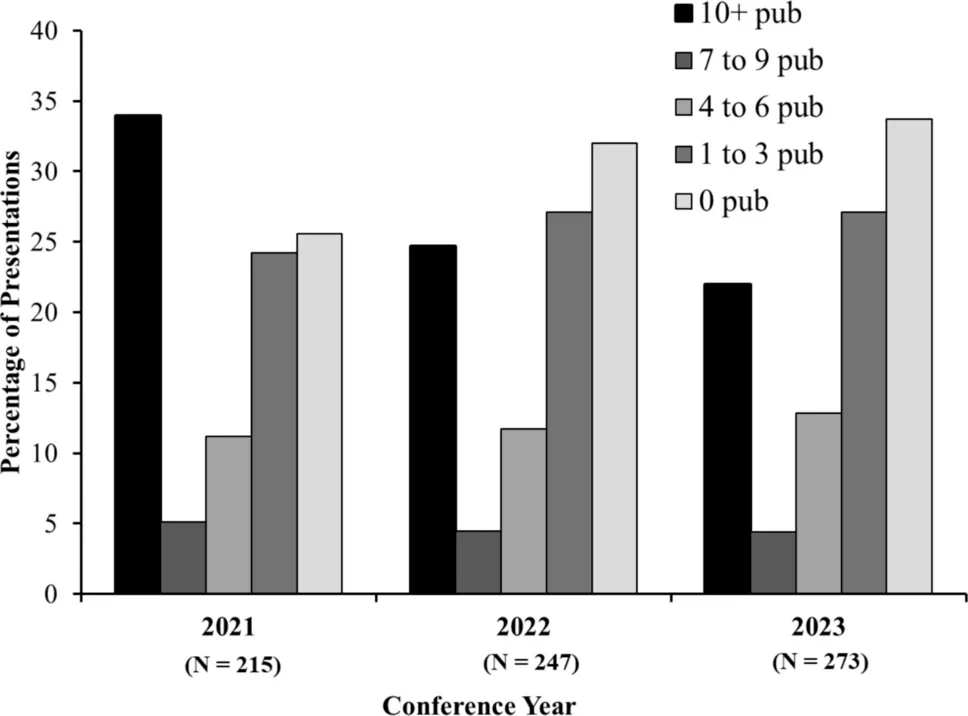
Percentage of speakers with 1–3, 4–6, and 7–9 topic specific publications across years. *Note*. The *Ns* for each year include all five topic-specific publication categories. Pub = topic-specific publication

Figure 4 depicts the percentage of keynote speakers and invited speakers with either 0 topic-specific publications or 10 + topic-specific publications across 2021, 2022, and 2023. We chose to depict these two publication-count designations as they represent the starkest contrast of the publication record continuum. Over the three years, there was a *decrease* in the percentage of speakers with 10 or more topic-specific publications and an *increase* in the percentage of speakers with 0 topic-specific publications. In addition, Fig. 5 shows a slight increase in the percentage of speakers with 1–3 and 4–6 topic-specific publications across the three years.

**Fig. 6:**
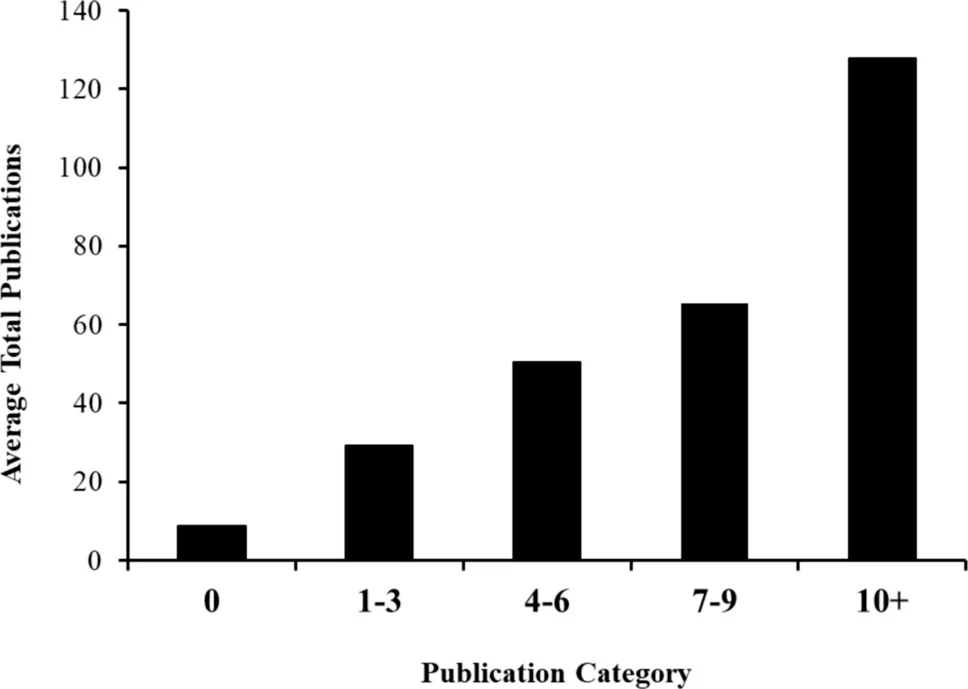
Total publication count by topic-specific publications for keynote and invited speakers. *Note*. For the 0 topic-specific publication category, 90 of the 226 presenters did not have any publications

Although the number of topic-specific publications on each speaker’s presentation topic served as the primary source for determining expertise, Fig. 6 depicts the general research expertise of invited speakers using the average publication count across the five topic-specific publication categories. Results indicate that keynote speakers and invited speakers with 0, 1–3, 4–6, 7–9, and 10 + topic-specific publications had an average of 8.76 (range, 0 to 256), 29.31 (range, 1 to 181), 50.45 (range, 4 to 181), 65.35 (range, 9 to 153), and 127.91 (range, 17 to 796) total publications, respectively. Regarding the 0 top-specific publication category, 90 of the 226 presenters did not have any publications. Thus, nearly 40% of the keynote speakers and invited speakers with 0 topic-specific publications had *not* published research in other areas. The evaluation of alternative reasons for organizations selecting the 226 keynote speakers and invited speakers with no topic-specific publications revealed that 85% of the speakers had one or two of the listed experiences (see Fig. 1), 8% of speakers had three of the listed experiences, and 4% of speakers did not have any of the listed experiences.

Table 1 shows the ABAI topic categories for all keynote and invited presentations across the 0, 1–3, 4–6, 7–9, and 10 + topic-specific publication categories. Across all ABAI topics, the highest percentage of all presentations fit the CSS/PCH (39.9%) topic category, followed by the AUT/DDA (22.3%) and TBA/OBM (15.6%) topic categories. For the 226 speakers with zero topic-specific publications, 127 (56.2%), 32 (14.2%), and 42 (18.6%) presentations were in the CSS/PCH, AUT/DDA, and TBA/OBM categories, respectively.

**Table 1:** ABAI categories of keynote and invited presentation across topic-specific publication count

Topic specific publications	CSS/PCH	AUT/DDA	TBA/OBM	EDC	CBM/BPM	EAB	DEV/VRB	AAB	Totals
0	127	32	42	12	5	1	7	0	226
1–3	86	44	22	10	10	11	10	0	193
4–6	32	18	17	4	7	5	4	1	88
7–9	5	17	6	1	1	1	3	0	34
10 +	43	53	28	21	19	20	8	2	194
Totals	293	164	115	48	42	38	32	3	735

*N* = 735 keynote and invited presentations. Autism/Developmental Disabilities (AUT/DDA), Applied Animal Behavior (AAB), Experimental Analysis of Behavior (EAB), Community, Social, and Sustainability Issues/Philosophical, Conceptual, and Historical Issues (CSS/PCH), Teaching Behavior Analysis/ Organizational Behavior Management (TBA/OBM), Clinical, Family, and Behavioral Medicine/Behavior Pharmacology and Neuroscience (CBM/BPN), Behavioral Development/Verbal Behavior (DEV/VRB), and Education (EDC)

**Fig. 7:**
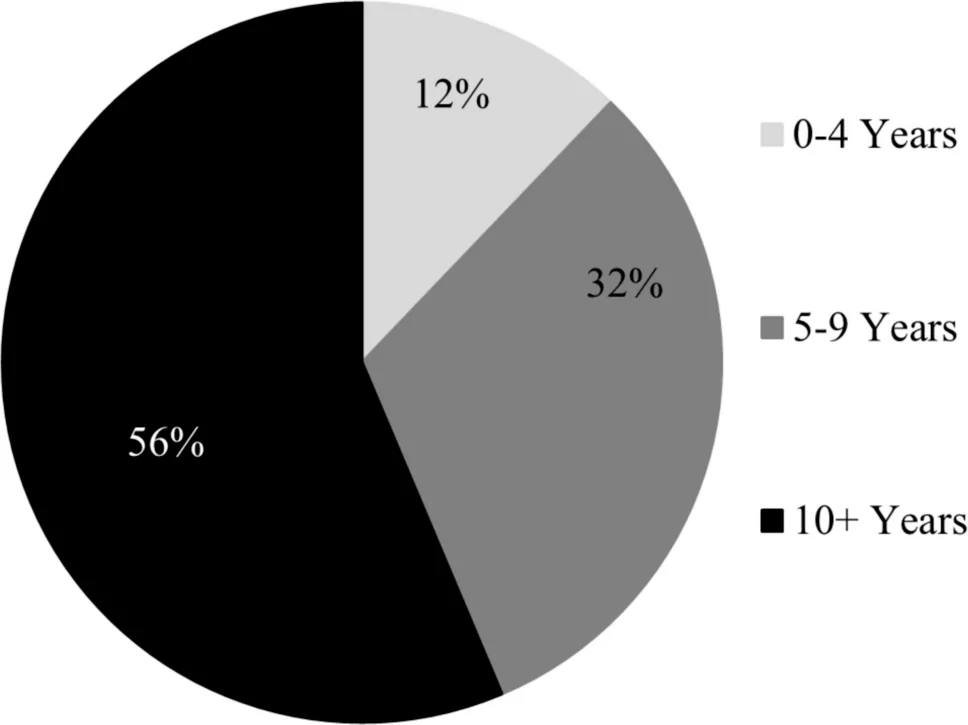
Years of experience of speakers with 0 publications who have a BCBA or BCBA-D certification. *n* = 181 board certified keynote speakers and invited presenters. Percentages were rounded to the nearest whole number

Figure 7 summarizes the *number of years certified* (only) for 181 board-certified presenters with 0 topic-specific publications. The majority (56.4%) of speakers had their BACB or BACB-D certification for over 10 years, while 22 speakers (12%) were certified for less than 5 years. Overall, we found that 80% of the speakers with 0 topic-specific publications who held BACB certification practiced with their certification for an average of 12 years (range, 2–23). Outside of an ABA background, we found 16 speakers with a related Ph.D. and 12 speakers from disciplines that related to their presentations.

## Discussion

This study evaluated a total of 735 invited presentations across 111 conferences during the years 2021, 2022, and 2023. Results indicated that nearly a third of keynote and invited speakers did not author *any* peer-reviewed publications related to their respective presentation topics. Concerningly, nearly 40% of speakers with 0 topic-specific publications did not have *any* publications. Results from our analysis of alternative reasons why organizations may have invited speakers with 0 topic-specific publications revealed that approximately 85% of these speakers had one or two alternative experiences.

Although conference committees may consider presentations on ethics and supervision unique cases, the content is not outside of the scope of ABA (e.g., see Brodhead et al., 2023) and should still be subjected to empirical evaluation (e.g., supervisee ratings of advisors who do and do not provide specific support, differential pass rates of graduates on the certification exam over time). Logic might suggest that the more years someone engages in a behavior, the more skilled they become. In one of the few studies that have employed this metric, Kuhlmann and Ardichvili (2015) found that more years in the practice of accounting did not necessarily give rise to the most skilled professionals within the applied discipline, which also requires CEUs. Their findings suggest the professional skill sets of people who engage in routine work may plateau when compared to those who engage in ongoing learning opportunities (i.e., consistently conduct and publish research). To this end, Kuhlmann and Ardichvili concluded that the quality of work experience was more crucial than the years of experience. Future research should evaluate whether BCBAs who have practiced for 10 or more years demonstrate more extensive skill sets in ABA than recently graduated practitioners.

The most obvious and immediate clinical implication of CEU presentations by speakers with no scientific expertise is the potential harm it can cause clients (e.g., Brodhead et al., 2018). Although the current study did not attempt to evaluate adverse effects on clients, direct harm could come to a client from a practitioner who implements a nonempirically supported procedure that either does not help or hurts the client (e.g., see Posey et al., 2024). At best, practitioners could avoid presentations by speakers who do not have expertise and thus mitigate some of this potential harm. For example, a practitioner who is considering a given conference could use Google to quickly determine whether the conference’s keynote speaker holds sufficient expertise in their presentation topic. Nevertheless, allocating organizational resources and time to support nonempirically based presentations necessarily displaces time and resources that could be used to support and disseminate empirically supported information. Thus, action at the organizational level is required to reverse the trend identified in this study (e.g., additional review and oversight by the ACE Coordinator).

A more distal clinical implication is a potential increase in the research-to-practice gap within behavior analysis. Over time, the trend identified in this study could result in a less scientific culture within the field and a corresponding decrease in effectiveness of practice. Or worse, clinicians may begin using nonempirically supported practices solely because they heard a compelling, non-data-based presentation on the topic. Future research should determine if the proportion of keynote and invited speakers without expertise identified through this study represents a cultural shift in the field by investigating conferences across a greater range of time. Further, future research could determine the extent to which the level of expertise expected for continuing education within the field of behavior analysis compares favorably to other fields of clinical practice.

There are at least three potential courses for reversing the trend of conferences inviting presenters without sufficient expertise in their presentation topics; these courses of action are not mutually exclusive. First, because practicing BCBAs are responsible for upholding the ethics code to protect their own certification and licensure, they should be accountable for selecting CEU opportunities that are grounded in peer-reviewed material (see Bank & Ingvarsson, 2024). This course of action does not eliminate the problem of CEUs presentations by non-experts, but it could decrease the number of BCBAs in attendance of those presentations. On the basis of our experiences organizing state conferences, it appears that some practitioners may be too willing to consume nonempirical but entertaining CEU content. Second, the BACB could alter the CEU requirements for the ACE providers. The current ACE provider handbook (BACB, 2022) stipulates that presenters must possess expertise on new subject matter as determined by the ACE provider’s review of the presenter’s CV. The handbook also stipulates that ACE providers must provide evidence of the instructor’s qualifications. Because the handbook does not specify what constitutes expertise on a given topic, some additional guidelines (e.g., requiring recent peer-reviewed publication on the topic) for ACE providers may be warranted. A third level of action involves setting standards to guide ABAI affiliated chapter leaders to invite presenters with sufficient expertise. To be sure, there are many barriers to the process of inviting speakers, but setting standards for committee members could result in a shift toward increasing the number of presentations with expert presenters. Searching a prospective speaker’s publications requires only minutes, and consumers or executive committees can decide on the qualifications of a speaker providing any form of CEU content.

Results from this study should be considered within the context of some limitations. First, we included some materials that were published *after* the invited speakers presented on the topic; this was done intentionally to allow for delays in publication. Thus, some speakers we scored as having 1–3 topic-specific publications may have had no publications on their presentation topic at the time of their presentation. This additional allowance, however, rarely affected the dependent variables. Second, the methods did not directly assess the delivery of information at the conference. For example, a presenter we categorized as an expert on their presentation content could have presented their material in a manner that was neither consumable for nor relevant to practitioners. In this way, the CEU presentation would fall short of the BACB (2022) expectations for a meaningful continuing education event. Nevertheless, such an assessment was beyond the scope of the present study. Third, some studies suggest that Google Scholar may not provide consistent information about researchers’ publications. For example, Dixon et al. (2015b) noted that Google Scholar occasionally provided differing publication counts for researchers on different days (for broader issues with Google Scholar, see García-Pérez, 2010 and Gusenbauer & Haddaway, 2020). Relatedly, the overall IOA score across all five categories for topic-related publications was somewhat low (i.e., 83%). Nevertheless, the IOA score for the 0 publications category (only), which was the primary focus of this study, was 93%. Thus, both coders typically identified keynote and invited presenters who had not published on their topics. Fourth, the current study did not assess why conference-selection committees invited speakers with no expertise to present at state ABA conferences. Notably, Kranak and Onofrio (2025) recently determined that many state ABA organizations selected their keynote and other invited speakers on the basis of the speaker’s popularity, which included how frequently the speaker presented at other conferences. Alternatively, we previously speculated that the rapid growth of the field, and the corresponding increase in the number of state chapters, has outpaced the availability of qualified speakers. Thus, conference committees may be opting for presenters without expertise because presenters with expertise are not available. Our measure of alternative experiences for presenters with no scientific experience yielded information that suggests speakers may have been selected due to self-dissemination of their clinical experience, self-published works, workshops, or a social media/podcast presence. Finally, the generality of the findings may be limited because the study covered years during which COVID-19 was highly prevalent. Thus, it is possible that junior researchers with less research expertise were more willing to present at face-to-face conferences than senior researchers who felt more susceptible to the virus. To this end, many of the conferences reviewed for this study were delivered online. Thus, we would not expect COVID-19 to differentially impact the participation of older presenters at conferences. Moreover, COVID-19 is likely to be an enduring rather than episodic phenomenon (e.g., Murray & Piot, 2021). Thus, what we learned about conference practices over the study time period is likely applicable thereafter.

Future studies could evaluate alternative modalities in which behavior analysts accrue CEU content. Our data on alternative sources indicated that the titles of some of the podcasts and webinars matched the titles of the invited speaker presentations identically. Future research could also evaluate the conference executive committee makeup and selection process in order to determine some indicators of people who invite content-expert speakers to the conferences. Without further analysis, on the one hand, we might predict that doctorate-level executive committee members not only value research-driven speakers but also have the network to invite other doctorate-level professionals to disseminate their research at conferences. On the other hand, we might speculate that younger up-and-coming professionals serving on committees might be more inclined to invite individuals to speak because they viewed their content on social media or heard them on a podcast, without vetting the extent to which they have documented expertise via publications. The mild disparity between the total publication count for speakers in the 0 topic-related publications category suggests future research should also evaluate the extent to which researchers with extensive publication records, but who either have not published articles in the past 5 years or have published on related topics, are serving as keynote or invited speakers at conferences.

In summary, the field of ABA has been based on pragmatic science and is thus expected to change in accordance with the available scientific evidence. The current study identified a potentially problematic trend with invited presentations at state-level ABA conferences. We believe that the trend identified in this study is reversible with the individual and organization-level actions outlined above.

## Data Availability

Data from this study are available from the corresponding author upon request.

## Footnotes

1 This includes doctoral level BCBAs (BCBA-Ds).

2 The BACB does not track this information (J. Carr, personal communication, August 8, 2024).

3 Richling et al. tallied the presentation as being published if one or more data sets from the presentation were published.

4 We distinguish between keynote speakers and other invited speakers because keynote speakers are typically recognized as having the highest status, and presumably expertise, of the presenters at a given conference (e.g., Kranak & Onofrio, 2025).

5 In part, this determination stipulated that the research process could not identify specific individuals, by name, in an evaluation process.

6 Including Puerto Rico, the total count is 55.

7 This method is a variation of the block-by-block method described by Mudford et al. (2009).
